# Complete Genome Sequences of Both Biotypes of a Virus Pair of Bovine Viral Diarrhea Virus Subgenotype 1k

**DOI:** 10.1128/genomeA.00287-13

**Published:** 2013-07-25

**Authors:** Adriano Marques Antunes de Oliveira, Hanspeter Stalder, Ernst Peterhans, Kay-Sara Sauter, Matthias Schweizer

**Affiliations:** Institute of Veterinary Virology, University of Bern, Bern, Switzerland

## Abstract

We determined the complete genome sequences of both biotypes of a virus pair of bovine viral diarrhea virus (BVDV) subgenotype 1k. The viruses were isolated from a persistently infected calf suffering from mucosal disease. Compared to the noncytopathic biotype, the cytopathic biotype contains an insertion of 84 nucleotides and 22 nucleotide changes.

## GENOME ANNOUNCEMENT

Bovine viral diarrhea virus (BVDV), including the two species BVDV-1 and BVDV-2, is part of the genus *Pestivirus* in the family *Flaviviridae*. BVDV-1 may be further subdivided into 11 to 16 subgenotypes (1, 2). BVDV exists in two biotypes, cytopathic (Cp) and noncytopathic (Ncp), depending on the effect on cultured cells. Solely Ncp-BVDV is able to generate persistently infected (PI) calves by invading the fetus early in gestation. Such PI calves are at risk of developing fatal mucosal disease (MD), when both biotypes can be isolated that are antigenically closely related, hence referred to as a “virus pair.” The Cp biotype differs from the Ncp virus by nucleotide substitutions or insertions of viral or cellular sequences (3–5).

The BVDV 1k subgenotype was first described in Switzerland (6–8). Of the BVDV-1 strains circulating in Switzerland, 21% belong to this subgenotype (9), whereas outside of Switzerland, only a few cases have been described in eastern Austria (10).

Here, we report the full-length sequences of both biotypes of a virus pair of the BVDV 1k subgenotype. The Ncp biotype, SuwaNcp, was isolated from the peripheral blood before the onset of MD, whereas its Cp counterpart, SuwaCp, was isolated after the onset of MD. Separation and biological cloning of the two biotypes were performed by plaque formation (6, 11, 12). The sequence of SuwaNcp was determined by four overlapping PCR fragments that were cloned into an appropriate vector (12), whereas the sequence of SuwaCp was directly determined from PCR fragments obtained after RNA isolation of infected bovine turbinate cells (QIAamp RNA blood minikit; Qiagen Hombrechtikon, Switzerland) and reverse transcription-PCR (RT-PCR) (Qiagen OneStep RT-PCR kit), according to the manufacturer’s instructions. The PCR fragments were purified with the QIAquick PCR purification kit (Qiagen), used according to the manufacturer’s instructions. DNA-Sanger cycle sequencing with BigDye Terminator chemistry (v3.1) and capillary electrophoresis (ABI 3730xl DNA analyzer; Applied Biosystems) was performed at Microsynth (Balgach, Switzerland). The electropherograms obtained were assembled with the SeqMan II sequence analysis software (v5.01; DNAStar, Inc., Madison, WI) and the sequences were analyzed with the Clone Manager 9 Professional Edition (Scientific & Educational Software, Cary, NC) and the MEGA program v5.05 (13).

The genome of SuwaNcp comprises 12,271 nucleotides (nt) and contains one large open reading frame (ORF) that encompasses 3,898 amino acids (aa). SuwaCp comprises 12,355 nt and one ORF with 3,926 aa. The 5′- and 3′-untranslated regions (UTR) of both viruses are 384 nt and 190 nt long, respectively. SuwaCp contains an insertion of 84 nt between nt 4368 and 4369 of the SuwaNcp. This insertion is an in-frame duplication of the sequences of nonstructural proteins NS4B (nt 7393 to 8433) and NS5A (nt 8434 to 9921). In addition, the sequences differ in 22 positions (18 transitions and 4 transversions), with only 9 of them leading to aa changes (four in NS2, one each in E^rns^, E2, NS3, NS4B, and NS5A). Interestingly, several insertions at the same locations were described previously. Thus, an insertion of cellular origin (14), or 27-nt and 45-nt insertions of viral sequences of the NS2 and the NS4B-NS5B junction regions were found in the BVDV-1b strain Cp7 and in several vaccine-derived viruses, respectively (14, 15). In accordance with these reports (15, 16), replacing the entire NS2 region of SuwaNcp with the corresponding region of SuwaCp yields a cytopathic virus. However, whether the insertion alone or only in combination with the point mutations in NS2 is responsible for the cp phenotype remains to be determined. Nevertheless, these results support a possible hot spot for recombinations at this location in NS2 (4, 14).

### Nucleotide sequence accession numbers.

The genomic sequences of strains SuwaNcp and SuwaCp have been deposited in GenBank under accession no. KC853440 and KC853441, respectively.
